# The role and predictive value of Th17/Treg imbalance and inflammatory factors in sagittal imbalance after VCD for ankylosing spondylitis

**DOI:** 10.5937/jomb0-58100

**Published:** 2025-10-28

**Authors:** Jin Du, Wang Tianhao, Chao Xue, Guoquan Zheng, Yan Wang

**Affiliations:** 1 First Medical Center of PLA General Hospital, Department of Orthopedics, Beijing, 100853, China

**Keywords:** ankylosing spondylitis, Th17/Treg, inflammatory factors, sagittal balance, vertebral column decancellation, ankilozirajući spondilitis, Th17/Treg, inflamatorni faktori, sagitalni balans, dekancelacija kičmenog stuba

## Abstract

**Background:**

This study aimed to explore the impact of T helper 17 (Th17)/regulatory T (Treg) cells imbalance and inflammatory factors (IL-1b, IL-18, and TNF-a) on sagittal balance in patients with thoracolumbar kyphosis due to ankylosing spondylitis (AS) following vertebral column decancellation (VCD), to clarify the predictive efficacy of the Th17/Treg ratio and IL-1b, IL-18, and TNF-a in post-operative sagittal imbalance.

**Methods:**

A total of 134 AS patients undergoing VCD were enrolled and categorised into an imbalance group (46 cases) and a balance group (88 cases), depending on post-operative sagittal balance. Measurements of the Th17/Treg ratio and inflammatory factor levels were conducted at three time points: preoperatively (T0), 24 hours postoperatively (T1), and 4 weeks postoperatively (T2). These data were analysed alongside sagittal parameters (SVA, PI-LL, and PT) to assess their correlations and predictive potential.

**Results:**

At 4 weeks after surgery, the imbalance group showed significantly elevated Th17/Treg ratios and higher levels of IL-1b, IL-18, and TNF-a compared to the balance group (P&lt;0.05). Th17/Treg ratios and inflammatory factors (IL-1b, IL-18, TNF-a) showed significant positive correlations with SVA, PI-LL, and PT (P&lt;0.05). Notably, the combined assessment of Th17/Treg ratio and inflammatory factors sagittal imbalance had a sensitivity of 52.17% and specificity of 90.91 (P&lt;0.001).

**Conclusions:**

The findings suggest that Th17/Treg imbalance and excessive expression of IL-1b, IL-18, and TNF-a are strongly linked to postoperative sagittal imbalance in AS patients. These biomarkers may serve as valuable early predictors for assessing surgical outcomes.

## Abstract (sr)

**Uvod:**

Ova studija je imala za cilj da ispita uticaj disbalansa T pomoćnih ćelija tipa 17 (Th17) i regulatornih T ćelija (T reg), kao i inflamatornih faktora (IL-1b, IL-18 i TNF-a) na sagitalni balans kod pacijenata sa torakolumbalnom kifozom usled ankilozirajućeg spondilitisa (AS) nakon dekancelacije kičmenog stuba (VCD), kako bi se pojasnila prediktivna efikasnost odnosa Th17/T reg i inflamatornih markera IL-1b, IL-18 i TNF-a u postoperativnom sagitalnom disbalansu.

**Metode:**

Ukupno 134 pacijenta sa AS koji su podvrgnuti VCD su podeljeni u grupu sa disbalansom (46 slučajeva) i grupu sa balansom (88 slučajeva), u zavisnosti od post operativnog sagitalnog balansa. Merenja odnosa Th17/Treg i nivoa inflamatornih faktora vršena su u tri vremenske tačke: preoperativno (T0), 24 sata nakon operacije (T1) i 4 nedelje nakon operacije (T2). Ovi podaci analizirani su zajedno sa sagitalnim parametrima (SVA, PI-LL i PT) radi procene njihove korelacije i prediktivnog potencijala.

**Rezultati:**

Četiri nedelje nakon operacije, grupa sa disbalansom je pokazala značajno povišene odnose Th17/Treg i više nivoe IL-1b, IL-18 i TNF-a u poređenju sa grupom sa sagitalnim balansom (P&lt;0,05). Odnos Th17/T reg i inflamatorni faktori (IL-1b, IL-18, TNF-a) pokazali su značajne pozitivne korelacije sa SVA, PI-LL i PT (P&lt;0,05). Ono što je bilo bitno je da je kombinovana procena odnosa Th17/Treg i inflamatornih faktora za sagitalni disbalans imala senzitivnost od 52,17% i specifičnost od 90,91% (P&lt;0,001).

**Zaključak:**

Rezultati ukazuju da su disbalans Th17/Treg ćelija i prekomerna ekspresija IL-1b, IL-18 i TNF-a snažno povezani sa postoperativnim sagitalnim disbalansom kod pacijenata sa AS. Ovi biomarkeri mogu predstavljati značajne rane pokazatelje za procenu ishoda hirurškog lečenja.

## Introduction

Ankylosing spondylitis (AS), a chronic immunemediated arthritis of unknown origin, is characterised by inflammation affecting the axial skeleton, peripheral joints, and entheses [1]. Unlike other systemic autoimmune disorders, AS is primarily driven by the innate immune system, which exhibits abnormal activity in both innate and innate-like immune cells [2]. In China, the prevalence of AS has been estimated at approximately 0.29%, as confirmed by a meta-analysis conducted by Zhao J et al. [3], with recent studies indicating a steady increase in its incidence [4]. The disease progression is marked by significant joint bone resorption, ultimately leading to spinal and lumbar kyphosis. This severely impairs patients’ ability to perform daily activities such as standing upright, lying flat, and maintaining a horizontal gaze [5]. Currently, vertebral column decancellation (VCD) is the standard treatment for AS, and its clinical efficacy has been widely acknowledged [6]. However, a subset of patients may develop postoperative sagittal imbalance due to factors such as undercorrection, overcorrection, or disruption of the spine-pelvic compensatory mechanism [7]. Sagittal imbalance, defined as the failure of the spine-pelvic complex to restore normal sagittal alignment, results in a shift of the body’s centre of gravity from its physiological range, leading to compensatory postures, such as forward trunk tilt and pelvic retroversion, as well as mechanical dysfunction. Hence, sagittal imbalance is now recognised as a key determinant of VCD outcomes [8]. Despite its significance, no effective strategies exist to prevent postoperative sagittal imbalance in AS patients.

The balance between T helper 17 (Th17) and regulatory T (Treg) cells plays a pivotal role in immune regulation [9]. A reduction in the number or function of Treg cells leads to an increase in Th17 cell count and activity, triggering the release of various inflammatory mediators and systemic inflammatory responses [10]. In the context of AS, a meta-analysis by Liu D et al. confirmed the presence of Th17/Treg imbalance [11]. Moreover, the research by Ding T et al. revealed that this imbalance is associated with cardiovascular complications in AS patients, further underscoring its clinical relevance [12]. These findings suggest that Th17/Treg balance could serve as a valuable biomarker for assessing AS progression. Equally important is the role of inflammatory mediators in AS. Dysregulation of several interleukin (IL) family members has been documented in AS [13], with evidence linking these changes to disease activity [14]. However, the relationship between Th17/Treg, IL-1β, and other inflammatory factors with sagittal imbalance in AS patients remains unexplored.

Achieving early assessment of postoperative sagittal imbalance represents a critical step toward enhancing the clinical outcomes of VCD. This study aims to elucidate the relationship between Th17/Treg, IL-1β, and other key immune and inflammatory factors in AS with sagittal balance, providing novel insights that could inform future clinical evaluation and management strategies.

## Materials and methods

### Study population

**Figure 1:**
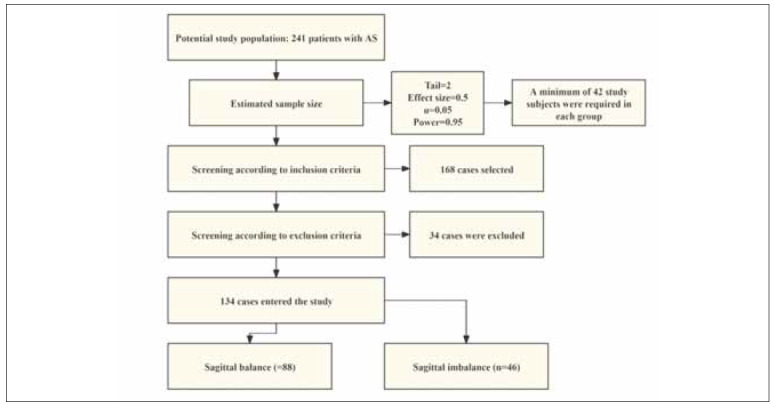
Screening process for research subjects.

A retrospective analysis was designed, with the sample size calculated using G*Power 3.1 to ensure statistical adequacy. A total of 134 AS patients were selected based on stringent inclusion and exclusion criteria. The VCD procedure was uniformly performed by a dedicated surgical team at our hospital following patient admission. The osteotomy segments were selected as L2–L4 and fixed with pedicle screws (5.5 mm in diameter and 45 mm in length). Of these, 46 patients were classified into the sagittal imbalance group due to postoperative imbalance following VCD surgery, while the remaining 88 patients were assigned to the sagittal balance group. Figure 1 illustrates the process of screening our research subjects. To minimise bias, neither the study subjects nor the data collectors were aware of the patient grouping.

Inclusion Criteria: Patients were included if they met the following conditions: (1) A confirmed diagnosis of AS accompanied by lumbar kyphosis. (2) Experience of receiving VCD during their hospital stay. (3) Comprehensive and complete clinical records. Exclusion Criteria: Patients were excluded from the study if they exhibited any of the following: (1) A prior history of spinal pathologies or related surgical interventions. (2) Lumbar kyphosis attributed to causes other than AS. (3) Significant leg length discrepancy. (4) Symptoms indicative of spinal cord or neurological involvement. (5) Concurrent conditions such as malignancies, immune system disorders, or psychiatric illnesses.

### Ethical considerations

The study protocol has received approval from our institution’s ethics committee. Prior to their inclusion in the study, all participants provided written informed consent. The research adhered strictly to the ethical guidelines set forth in the Declaration of Helsinki, safeguarding the rights and well-being of all participants.

### Baseline data collection

Key indexes, including patients’ age, gender, duration of AS, pathological stage of AS, operation time, intraoperative blood loss, and length of postoperative hospital stay, were comprehensively collected.

### Assessment of sagittal balance

To evaluate sagittal balance, full-spine lateral radiographs (Siemens Axiom Aristos VX, Germany) were acquired at two time points: preoperatively and 4 weeks postoperatively. The voltage is 70 kV, the current is 200 mA, and the shooting distance is 1.5 meters. Key parameters measured included the sagittal vertical axis (SVA), pelvic incidence (PI), lumbar lordosis (LL), and pelvic tilt (PT). The sagittal imbalance was diagnosed if any of the following criteria were met: SVA>5 cm, PI-LL>10°, and PT>25° [15]. SVA and PI-LL were tested only preoperatively and 4 weeks postoperatively because they reflect long-term sagittal balance.

### Laboratory testing

Blood samples were collected from patients under fasting conditions before surgery (T0), 24 hours after surgery (T1), and 4 weeks after surgery (T2). Each sample was divided into two aliquots. One copy was used for the flow cytometer assay: heparin anticoagulated peripheral blood and Ficoll density gradient centrifugation to separate peripheral blood mononuclear cells (PBMC). PMA (50 ng/mL), ionomycin (1 μg/mL), and brefeldin A (1:1000) were added, and Th17 was stimulated with 5% CO at 37°C (4–6 h); Treg did not require stimulation. Anti-CD4-FITC, CD25-APC were incubated for 20 min at room temperature away from light. Treat with precooled fixative/film-breaker to detect intracellular labelling. Th17 with anti-IL-17A-PE, Treg with anti-FoxP3-PE-Cy7 for 30 minutes at 4°C protected from light. Data were acquired by flow cytometry (BD FACSCanto II, USA), and a gating strategy was set: IL-17A for Th17 and CD25 FoxP3 for Treg in CD4 cells. Antibodies were purchased from Abcam (USA). The other copy was used for enzyme-linked immunosorbent assay (ELISA): serum was separated by centrifugation (3000 rpm/min) for 15 min, and serum levels of IL-1β, IL-18, and tumour necrosis factor-α (TNF-α) were measured according to the kit instructions. The kits were purchased from Wuhan AmyJet Technology Co. All experiments were repeated three times to ensure data reliability. Th17/Treg and inflammatory factors were assayed at T0, T1, and T2 time points to assess the postoperative immune-inflammatory response dynamically.

### Outcome measures

The study focused on several key outcomes: (1) changes in sagittal balance parameters before and after surgery, (2) variations in Th17/Treg ratios and inflammatory factor levels, and (3) the predictive capacity of Th17/Treg and inflammatory factors for identifying postoperative sagittal imbalance.

### Statistical analysis

Data analysis was conducted using SPSS 26.0. Categorical variables, such as gender and pathological stage, were compared using the chi-square test. For continuous variables, including age and Th17/Treg ratios, normality was assessed using the Shapiro-Wilk test. Normally distributed data were analysed using independent samples t-tests and paired t-tests. A Bonferroni correction was used to control for one type of error in multiple comparisons. Non-normally distributed data were analysed using non-parametric tests, including Mann-Whitney U tests, Wilcoxon signed-rank tests, and Kruskal-Wallis H tests. Diagnostic performance was evaluated using receiver operating characteristic (ROC) curve analysis, while correlations were assessed using Pearson’s correlation coefficient. A *P*-value of less than 0.05 was considered statistically significant.

## Results

### Comparison of baseline data

The two groups demonstrated no significant differences in baseline data such as age, gender, and duration of AS (*P*>0.05), ensuring their comparability. Furthermore, operation time, intraoperative blood loss, and length of hospital stay were similar between the groups (*P*>0.05), indicating that these variables were not directly linked to sagittal balance outcomes (Table 1).

**Table 1:** No difference in baseline information between the two groups of study participants.

	Sagittal balance<br>(n=88)	Sagittal imbalance<br>(n=46)	χ^2^ or t-values	P-values
Sex			0.278	0.598
Male/female	52/36	25/21		
Age (years)	42.42±10.79	40.04±9.13	1.274	0.205
Duration of disease (years)	2.80±1.02	2.85±1.09	0.275	0.784
Pathologic grade of AS			0.671	0.413
I/II	59/29	34/12		
Operating time (min)	267.76±18.02	270.07±25.71	0.604	0.547
Intraoperative bleeding (mL)	1058.55±77.29	1073.26±91.17	0.983	0.327
Length of hospital stay (d)	15.07±2.03	15.35±2.51	0.697	0.487

### Changes in sagittal balance parameters before and after surgery

Preoperative measurements of SVA, PI-LL, and PT showed no significant differences between the two groups (*P*>0.05). At the 4-week postoperative follow-up, both groups exhibited notable improvements in SVA, PI-LL, and PT (*P*<0.05). However, the balanced group achieved significantly better outcomes in these parameters compared to the imbalanced group (*P*<0.05) (Table 2).

**Table 2:** Postoperative sagittal balance parameters were lower in the sagittal balance group.

		Sagittal balance<br>(n=88)	Sagittal imbalance<br>(n=46)	t-values	P-values
SVA (cm)	Preoperative	22.24±7.06	22.56±4.94	0.275	0.784
	Postoperative	9.80±3.16	13.60±5.06	5.328	<0.001
	t-values	15.090	8.595		
	P-values	<0.001	<0.001		
PL-LL (°)	Preoperative	52.06±18.16	49.36±6.51	0.823	0.412
	Postoperative	14.97±5.41	19.13±6.51	3.938	
	t-values	18.362	10.893		
	P-values	<0.001	<0.001		
PT (°)	Preoperative	39.63±11.47	39.59±9.86	0.019	0.985
	Postoperative	28.91±8.45	33.49±9.06	2.905	0.004
	t-values	7.055	3.090		
	P-values	<0.001	0.003		

### Changes in Th17/Treg and inflammatory factors before and after surgery

At T0, Th17/Treg ratios and levels of IL-1β, IL-18, and TNF-α were comparable between the two groups (*P*>0.05). By 24 hours postoperatively (T1), both groups experienced significant declines in Th17/Treg ratios and inflammatory factor levels compared to baseline (*P*<0.05), and the sagittal imbalance group was higher than the sagittal balance group (*P*<0.05). At 4 weeks postoperatively (T2), further reductions in Th17/Treg ratios and inflammatory markers were noted in both groups compared to T0 and T1 (*P*<0.05). Importantly, the balance group displayed significantly lower values than the imbalance group at T2 (*P*<0.05) (Table 3).

**Table 3:** Postoperative Th17/Treg and inflammatory factors were lower in sagittal balance group.

		Sagittal balance<br>(n=88)	Sagittal imbalance<br>(n=46)	t-values	*P*-values
Th17/Treg	T0	2.53±0.29	2.62±0.41	1.495	0.137
	T1	1.93±0.39	2.10±0.35	2.488	0.014
	T2	0.80±0.23	1.30±0.22	12.274	<0.001
	F-values	723.713	181.107		
	P-values	<0.001	<0.001		
IL-1β (pg/mL)	T0	6.60±1.15	6.35±1.10	1.196	0.234
	T1	5.33±0.78	5.90±1.10	3.478	0.009
	T2	4.18±0.53	4.56±0.84	3.305	0.001
	F-values	176.342	38.352		
	P-values	<0.001	<0.001		
IL-18 (pg/mL)	T0	9.21±1.47	9.44±1.47	0.860	0.392
	T1	7.92±1.12	8.47±1.00	2.834	0.005
	T2	4.29±0.79	5.35±1.01	6.676	<0.001
	F-values	425.618	151.083		
	P-values	<0.001	<0.001		
TNF-α (ng/mL)	T0	104.75±16.70	106.69±18.91	0.609	0.544
	T1	83.02±8.90	88.11±12.52	2.725	0.007
	T2	56.03±7.38	68.01±8.97	8.282	<0.001
	F-values	381.024	86.826		
	P-values	<0.001	<0.001		

### Predictive value of Th17/Treg and inflammatory factors for sagittal imbalance

**Figure 2:**
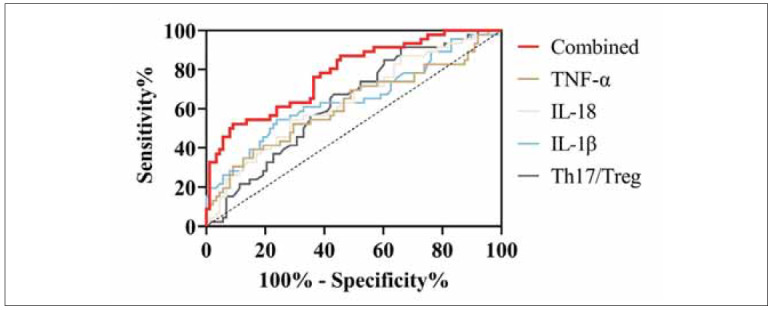
ROC curves for Th17/Treg ratio (T helper 17/regulatory T cells) and inflammatory factors (IL-1β, IL-18, TNF-α) in predicting sagittal imbalance.

ROC curve analysis was performed using Th17/Treg ratios, and levels of IL-1β, IL-18, and TNF-α measured at T1 to assess their predictive utility for sagittal imbalance. The results highlighted the strong predictive performance of these biomarkers, with the IL-1β ratio achieving an AUC of 0.650. When combined, Th17/Treg and inflammatory factors demonstrated a sensitivity of 52.17% and specificity of 90.91% for identifying sagittal imbalance (*P*<0.05) (Table 4 and Figure 2).

**Table 4:** Diagnostic effect of Th17/Treg and inflammatory factors on sagittal imbalance.

Logistic regression analysis					
	Th17/Treg	IL-1b	IL-18	TNF-a	Combined
B	1.346	0.774	0.641	0.046	-16.895
S.E. 0.558	0.233	0.221	0.021	3.383	
Wals	5.808	11.066	8.432	4.754	24.940
OR	3.841	2.169	1.898	1.047	-
95%CI	1.286–4.473	1.374–3.423	1.232–2.926	1.005–1.091	-
*P*	0.016	0.001	0.004	0.029	<0.001
Diagnostic effect					
	Th17/Treg	IL-1b	IL-18	TNF-a	Combined
Cut-off	>1.755	>5.785	>8.400	>90.410	>0.538
AUC	0.632	0.65	0.641	0.618	0.779
95%CI	0.537–0.728	0.547–0.752	0.542–0.740	0.512–0.723	0.698–0.861
Sensitivity (%)	91.3	54.35	54.35	39.13	52.17
Specificity (%)	34.09	76.14	71.59	84.09	90.91
*P*	0.012	0.005	0.008	0.026	<0.001

The AUC for the diagnosis of sagittal imbalance was significantly higher using the combination of Th17/Treg and inflammatory factors compared to single indicators. Table 5

**Table 5:** Correlation of Th17/Treg, inflammatory factors and sagittal parameters

		Th17/Treg	IL-1β	IL-18	TNF-α
Preoperative	SVA	0.765/<0.001	0.681/<0.001	0.800/<0.001	0.808/<0.001
	PL-LL	0.531/<0.001	0.537/<0.001	0.659/<0.001	0.675/<0.001
	PT	0.750/<0.001	0.666/<0.001	0.683/<0.001	0.720/<0.001
	SVA	0.773/<0.001	0.860/<0.001	0.880/<0.001	0.621/<0.001
	PL-LL	0.690/<0.001	0.768/<0.001	0.827/<0.001	0.692/<0.001
	PT	0.780/<0.001	0.717/<0.001	0.769/<0.001	0.565/<0.001
Postoperative	SVA	0.657/<0.001	0.709/<0.001	0.540/<0.001	0.700/<0.001
	PL-LL	0.551/<0.001	0.767/<0.001	0.592/<0.001	0.742/<0.001
	PT	0.430/0.003	0.861/<0.001	0.645/<0.001	0.648/<0.001
	SVA	0.806/<0.001	0.557/<0.001	0.646/<0.001	0.796/<0.001
	PL-LL	0.801/<0.001	0.691/<0.001	0.715/<0.001	0.741/<0.001
	PT	0.646/<0.001	0.689/<0.001	0.752/<0.001	0.639/<0.001

### Correlation between Th17/Treg, inflammatory factors, and sagittal balance parameters

According to Pearson correlation analysis, Th17/Treg ratios and levels of IL-1β, IL-18, and TNF-α demonstrated significant positive correlations with sagittal balance parameters (SVA, PI-LL, and PT) both preoperatively and at 4 weeks postoperatively (*P*<0.05). These findings underscore the close relationship between immune-inflammatory markers and sagittal balance in AS patients.

## Discussion

This study revealed a significant association between Th17/Treg ratios, inflammatory factors (IL-1β, IL-18, and TNF-α), and sagittal balance parameters, offering novel perspectives for assessing postoperative recovery in AS patients undergoing VCD.

The comparability of the two groups was confirmed through the analysis of baseline data, with no significant differences observed in demographic or clinical factors. Furthermore, surgical-related indicators, such as operation time and intraoperative blood loss, were similar between the groups, indicating that sagittal imbalance is unlikely to be directly influenced by the surgical process itself. Postoperatively, both groups demonstrated improvements in SVA, PI-LL, and PT, reflecting enhanced sagittal alignment. However, the imbalance group consistently exhibited higher SVA, PI-LL, and PT values compared to the balance group. Clinically, postoperative sagittal imbalanceis thought to arise from multiple factors, including [1] suboptimal or excessive osteotomy correction (e.g., single-segment osteotomy may be insufficient for severe kyphosis), inadequate restoration of LL-PI matching, or inappropriate selection of fixation segments [15] [16].

Additionally, the intrinsic nature of AS contributes significantly. Patients with AS often experience spinal rigidity and a loss of compensatory mechanisms, leading to biomechanical chain disruption after surgery. Moreover, concurrent hip joint rigidity or flexion contractures can further increase the compensatory burden on the pelvis [17]. Despite these insights, the precise mechanisms underlying sagittal imbalance remain poorly understood.

In this study, we observed a consistent postoperative decline in Th17/Treg ratios and levels of IL-1β, IL-18, and TNF-α among AS patients following VCD. Notably, at 4 weeks post-operation, the imbalance group displayed elevated levels of these biomarkers compared to the balance group, with strong correlations to sagittal balance parameters. These findings suggest a potential interplay between Th17/Treg imbalance, inflammatory factors, and both disease progression and sagittal imbalance in AS. We suggest that the mechanisms by which Th17/Treg and inflammatory factors are involved in sagittal imbalance may have the following 3 points: (1) Imbalance drives the inflammatory-osteogenic vicious cycle. Supporting this notion, Xie J et al. reported that Sema4D induces Th17/Treg imbalance by activating the aryl hydrocarbon receptor in AS [18]. This finding aligns with our results and reinforces the role of Th17/Treg in AS pathogenesis. Moreover, Th17 cells contribute to ectopic osteophytes by secreting IL-17, which directly stimulates osteoblast differentiation and RANKL expression.

In contrast, Treg cell dysfunction results in inadequate suppression of inflammation, creating a detrimental cycle of »pro-inflammatory and pro-osteogenic« activity [19]. Additionally, Treg cells secrete IL-10 and TGF-β to modulate excessive immune responses, but their dysfunction may perpetuate chronic inflammation and abnormal bone remodelling [20]. We hypothesise that this imbalance not only exacerbates spinal rigidity but may also indirectly compromise sagittal balance by impairing postoperative bone healing, potentially leading to complications such as pseudarthrosis formation.

(2) TNF-α inhibits bone fusion. The maintenance of postoperative sagittal balance is critically dependent on the biomechanical compatibility of the spine-pelvis complex. In AS patients, the mechanisms underlying postoperative imbalance are intricately associated with the sustained influence of inflammatory factors [21]. TNF-α, a central pro-inflammatory mediator in AS, not only contributes to the destruction of spinal structures preoperatively but may also hinder postoperative mechanical reconstruction. This occurs through mechanisms such as inhibiting bone fusion (e.g., delaying healing at osteotomy sites) and inducing muscle atrophy, which compromises core stability [22]. Supporting this, Xu HW et al. [23] demonstrated that patients with elevated preoperative TNF-α levels exhibit a significantly higher likelihood of SVA correction failure, potentially due to a mismatch between PI and LL. (3) IL-1β/IL-18 synergistically promotes heterotopic ossification. IL-1β and IL-18 may disrupt sagittal alignment by promoting fibrosis and heterotopic ossification, altering stress distribution in the osteotomy region. This can lead to complications such as proximal junctional kyphosis or implant failure, further destabilising spinal alignment [24]. Moreover, the synergistic actions of IL-1β and IL-18 amplify the inflammatory cascade: IL-1β drives ligament ossification via the NF-B pathway, while IL-18 exacerbateslocal inflammation by enhancing the activity of Th1 cells and natural killer (NK) cells [25]. These inflammatory processes, coupled with Th17/Treg imbalance, collectively contribute to irreversible changes in spinal alignment, elevating the risk of sagittal imbalance following surgical correction.

Based on the mechanisms above, we propose that perioperative immune modulation could play a pivotal role in optimising outcomes for AS patients. Future strategies might include the preoperative use of biologics, such as TNF-α inhibitors or IL-17 antagonists, to reduce inflammatory burden and restore Th17/Treg balance, thereby improving bone healing quality. Additionally, VCD surgery should incorporate advanced osteotomy techniques, such as multilevel pedicle subtraction osteotomy, to achieve optimal LL-LLPI matching (target PI-LL 10°) while avoiding overcorrection that could result in pelvic retroversion (PT>25°). The strong correlations observed between Th17/Treg, IL-1β, IL-18, TNF-α, and sagittal balance parameters further suggest the potential for using these biomarkers to assess and predict sagittal imbalance dynamically. Our findings indicate that the combined evaluation of Th17/Treg, IL-1β, IL-18, and TNF-α at 24 hours postoperatively achieves a sensitivity of 52.17% and specificity of 90.91% for identifying sagittal imbalance, offering valuable clinical utility. This approach provides a promising strategy for preventing sagittal imbalance following VCD, with the potential to enhance long-term patient outcomes significantly.

Notwithstanding, this study, while elucidating the association between immune factors, inflammatory factors, and sagittal plane balance following VCD surgery, is not without its limitations: (1) The majority of evidence is derived from basic research and retrospective clinical data, necessitating validation through prospective cohort studies; (2) Genetic factors, such as HLA-B27 subtypes, which may influence immune response patterns, were not incorporated into the analysis. Future research should focus on multicenter cohort studies to develop predictive models that integrate preoperative immune markers (e.g., Th17/Treg ratios) with postoperative sagittal parameters. Additionally, the therapeutic potential of targeting the IL-1β/IL-18 pathway to improve postoperative mechanical stability warrants further exploration. There is also a focus that cannot be ignored, namely, the high cost of Th17/Treg with inflammatory factor assays, which may limit application in primary care. Therefore, in the future, we need to develop cost-effective alternative markers to optimise the prognosis of AS patients.

## Conclusion

Th17/Treg imbalance and the overexpression of IL-1β, IL-18, and TNF-α represent the shared pathological foundation for spinal rigidity and post-VCD sagittal imbalance in AS patients. By integrating perioperative immune microenvironment modulation with precise biomechanical reconstruction, it may be possible to overcome the current limitations of AS corrective surgery, thus improving long-term functional outcomes in patients.

## Dodatak

### Availability of data and materials

Original data in this study are available from the corresponding author upon reasonable request.

### Acknowledgements

Not applicable.

### Conflict of interest statement

All the authors declare that they have no conflict of interest in this work.
